# Pseudoachondroplasia: A rare cause of rhizomelic dwarfism

**DOI:** 10.4103/0019-5413.43400

**Published:** 2008

**Authors:** Anupama Tandon, Satish K Bhargava, Sandeep Goel, Shuchi Bhatt

**Affiliations:** University College of Medical Sciences and Guru Tegh Bahadur Hospital, Delhi, India

**Keywords:** Pseudoachondroplasia, rhizomelic dwarfism, skeletal dysplasia

## Abstract

Pseudoachondroplasia is a rare rhizomelic short-limbed skeletal dysplasia. Its inheritance is varied; autosomal dominant pattern and germline or somatic mutations can occur. Children at 2–3 years of age present with short height, gait disturbances, or limb deformities. Characteristic skeletal changes include shortening of long bones, predominantly of femur and humerus with irregular, flared metaphysis and fragmented epiphysis. Platyspondyly is also present, but the interpedicular distance is normal. The diagnosis is essentially based on imaging, and thus, it is important to be aware of the radiological features. Here, we report a case of two brothers where the elder sibling had classical radiological features of pseudoachondroplasia, whereas the younger one had early changes of this disorder.

## INTRODUCTION

Pseudoachondroplasia (PSACH) is a rare form of short-limbed dwarfism with a reported prevalence of approximately four per million individuals.^1^ Autosomal dominant inheritance has been reported in most cases.^2^ Ferguson *et al*, suggested somatic or germline mosaicism in some cases.^3^ Usually children at 2–3 years of age presents with delay in walking or waddling gait.

We report a case of two brothers where the elder sibling (8 years old) had classical radiological features of pseudoachondroplasia, while the younger sibling (2 years old) had early changes. Interestingly, their parents were of normal height, thus suggesting a somatic or germline mutation.

## CASE REPORT

An 8-year-old boy presented with complaints of short stature and an abnormal gait. According to his parents, the boy was normal until 3 years of age when he developed waddling gait, deformity of lower limbs, and retarded growth. The younger sibling was also normal since birth but had started developing waddling gait around 2 years of age.

**Figure 1:**
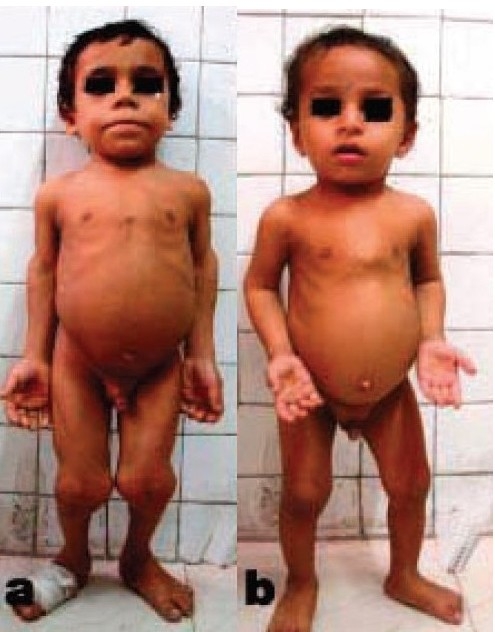
Clinical photograph of both male siblings (a- 8 year old, b- 2 year old) reveals rhizomelic shortening, genu valgum, exaggerated lordosis, and normal facies

Physical examination of the elder child revealed markedly reduced height measuring only 94 cm (<3rd percentile for age). Both the upper and lower segment length was reduced, but the limbs were disproportionately shortened. The upper segment measured 59 cm (mean for age is 65.4 cm), while the lower segment measured 35 cm (mean for age is 60 cm).^4^ The ratio of the length of the arm to forearm compartment was 0.62 and that of thigh to leg compartment was 0.69. In addition, there was limited elbow extension (up to 130° only), genu valgum deformity, and exaggerated lumbar lordosis. Systemic examination and intelligence were normal [Figure 1a].

The younger child was 83 cm tall (50th percentile of age is 84.10 cm) with a upper segment length of 50 cm (normal mean for age is 51.3 cm) and leg length of 33 cm (mean for age is 34 cm)^4^ and an exaggerated lumbar lordosis. Systemic examination and intelligence were normal [Figure 1b].

The parents were of normal height and intelligence. They, however, had a consanguineous marriage. There was no family history of dwarfism. A skeletal survey of both siblings was obtained.

**Figure 2:**
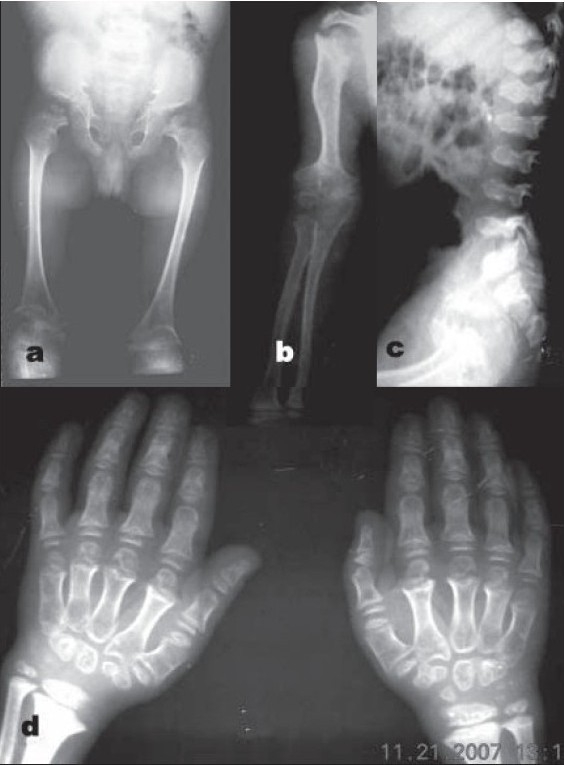
8 year old (elder) sibling. (a) Radiograph (anteroposterior view) of pelvis reveals squared ilium, narrow sacrosciatic notches, dysplastic acetabuli, and a characteristic medial beak at femoral neck. (b) Radiograph (anteroposterior view) of upper limb showing markedly flared and irregular metaphysis with deformed, irregular, and fragmented epiphyses. (c) Lateral radiograph of Lumbosacral spine showing platyspondyly with central beaking. (d) Radiograph (anteroposterior view) of hand shows underdeveloped carpals with short and broad metacarpals and phalanges

In elder child, the radiograph of pelvis revealed broad and squared iliac wings, narrow sacrosciatic notches, and dysplastic acetabuli with horizontal roofs. Both femora and humeri were disproportionately shortened with a characteristic medial beak at femoral neck [Figure 2a]. Metaphysis of all long bones were markedly flared and irregular with deformed, irregular, and fragmented epiphyses [Figure 2(a,b)]. The lumbosacral spine [Figure 2c] revealed thoracolumbar platyspondyly with normal interpedicular distance. Carpals were underdeveloped with short and broad metacarpals and phalanges [Figure 2d]. Skull radiographs, however, were normal.

**Figure 3:**
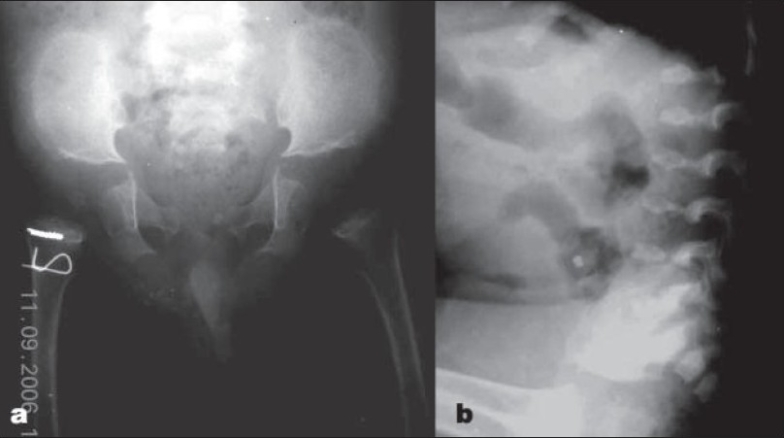
2 year old (younger) sibling. (a) Radiograph (anteroposterior view) of pelvis (in younger child) showing milder changes compared with that seen in elder sibling. (b) Radiograph (Lateral view) of Lumbosacral spine (in younger child) showing platyspondyly

In the younger child, the pelvic radiograph revealed squared iliac wings with dysplastic acetabuli and underdeveloped femoral epiphysis. The long bone shortening and severe metaphyseal changes seen in the elder sibling had not yet developed. The spine revealed mild platyspondyly [Figure 3].

Based on clinicoradiological features, a diagnosis of pseudoachondroplasia was made with classical features in elder child and early manifestations of this uncommon disorder in younger sibling.

## DISCUSSION

PSACH is a rare rhizomelic type of skeletal dysplasia which develops secondary to a mutation within genes encoding for cartilage oligomeric matrix protein (COMP) on chromosome.^19^ COMP is found in the extracellular matrix of the cartilage, tendon, and ligaments.^2^

Most families have demonstrated an autosomal dominant inheritance pattern, but in some cases, germline or somatic mutations have been suggested. In our case also, the parents were normal with two affected siblings, thus indicating probability of germline or somatic mosaicism in one of the parent.^2^,^3^

The children are invariably normal at birth, and they usually present either around 2 years of age with a delay in walking or a little later with an abnormal waddling gait or lower limb deformity. Subsequently, over the years, rhizomelic type of dwarfism becomes apparent with progressively increasing morbidity.

Physical examination reveals normal facies and intelligence. The adult height usually ranges between 82–130 cm with marked shortening of limbs. Associated deformities include genu valgum/varus, genu reccurvatum, limited elbow extension, kyphoscoliosis or increased lumbar lordosis, and joint laxity with secondary osteoarthritic changes.

The radiographic features^2^ include dramatically shortened bones, proximal more than distal, suggesting rhizomelic type of dwarfism, with flared and irregular metaphysis. Epiphyses are small, irregular, and often fragmented with delayed appearance, and the femoral capital and humeral epiphysis are most affected. Medial beaking of the femoral neck is one of the characteristic features (also present in our case). The hand and foot bones (metacarpals, metatarsals and phalanges) are broad and shortened with small and rudimentary epiphysis. Pelvis appears squared with broad iliac wings and narrow sacrosciatic notches. The acetabulum is poorly formed with horizontal roofs. The skull and facial bones are normal. Platyspondyly, anterior “beaking,” persistent oval shape, odontoid dysplasia, and disc space widening may also be present. Interpedicular distance is characteristically normal.

Differential diagnoses for this radiographic appearance in context of clinical findings include: achondroplasia, morquio syndrome, hypothyroidism, multiple epiphyseal dysplasia (MED), and spondyloepiphyseal dysplasia (SED) congenita.^5^

Achondroplasia patients have a large head with prominent frontal bones and a narrow base. The interpedicular distance decreases caudally in lumbar region but with normal vertebral height. The pelvis is square with small sciatic notches and shows classic champagne glass appearance. PSACH patients, on the other hand, have a normal skull and interpedicular distance with marked platyspondyly.

In MED, epiphyses are abnormal, but there are near normal metaphysis, pelvis, and spine unlike in PSACH, where metaphyseal and spinal changes are more marked. In SED, congenital epiphyseal changes mimic PSACH; however, spinal changes are more pronounced with marked kyphoscoliosis. Hip joints are affected disproportionately in relation to nearly normal distal limbs.

In Morquio syndrome, the spinal changes are prominent with flat vertebrae, central beaking, and marked kyphosis. Metacarpals show proximal tapering with short, wide tubular bones. Epiphysis may be affected, but metaphyseal widening and irregularity as seen in PSACH is absent.

In hypothyroidism, epiphyseal changes may simulate PSACH, but the dwarfism is symmetrical involving all long bones with slender shafts and endosteal scalloping. Metaphyses are normal. The skull shows wormian bones, J-shaped sella in young children, and cherry sella in older children. Bullet-shaped lumbar vertebra with kyphosis is seen, but general platyspondyly is lacking. Classical pelvis changes of PSACH are also lacking.^6^

Most patients with PSACH have early osteoarthritic changes and joint deformities requiring orthopedic intervention. COMP altering therapy can be used to treat this condition at genetic level.

## CONCLUSION

PSACH is a rare form of osteochondrodysplasia where patients invariably have skeletal complications because of early degenerative joint disease. Early recognition and diagnostic precision is essential for accurate prognostication, meaningful genetic counseling, and early therapy for joint manifestations.
